# Cognitive and Psychological Transfer Effects of Length-Dependent Working Memory Training in Healthy Older Adults

**DOI:** 10.3390/jintelligence14070124

**Published:** 2026-07-01

**Authors:** Caterina Padulo, Anna Cascone, Francesco De Crescenzo, Onofrio Gigliotta, Beth Fairfield

**Affiliations:** Department of Humanities, University of Naples Federico II, Via Porta Di Massa, 1, 80133 Naples, Italy; annacascone.anna@gmail.com (A.C.); francesco.decrescenzo@unina.it (F.D.C.); onofrio.gigliotta@unina.it (O.G.); beth.fairfield@unina.it (B.F.)

**Keywords:** working memory training, training length, transfer effects, successful aging, subjective health perception

## Abstract

The verbal working memory training proposed by Borella and co-authors found specific and transfer effects among older adults. However, the effective training lengths needed to maximize transfer effects are not yet clear. Also, far-transfer effects related to psychological well-being and subjective health are still under debate. The present study aimed to assess gains and transfer effects of a modified version of the WM training protocol by Borella and co-authors by comparing the original three-session (1 h each) version to a modified eighteen-session (1 h each) version. Our results confirmed the already demonstrated specific cognitive effects that seem to increase as the number of sessions increases. Regarding psychological well-being and subjective health, we found that while even three sessions of training can diminish reported loneliness and negative affective states, the longer training significantly improves the subjective perception of general health, suggesting that longer working memory training may be particularly fruitful in promoting well-being and successful aging.

## 1. Introduction

As global demographics increasingly shift toward an older population, the management of age-related cognitive decline has emerged as a paramount public health concern. Traditionally, intervention approaches have primarily emphasized “maintenance”—the preservation of existing functional levels to delay the onset of cognitive impairment. In contrast, current research advocates for a shift toward “optimization,” leveraging latent brain plasticity to enhance cognitive functioning and resilience. Within this context, working memory (WM) training interventions hold a crucial role since WM optimization may generate cascading benefits across a variety of complex daily activities and training the ability to maintain and manipulate information may also strengthen the underlying neural pathways and ultimately enhance brain plasticity and cognitive capacity.

Working Memory (WM) (Baddeley & Hitch, 1974), is a memory system responsible for the temporary storage and manipulation of information during ongoing tasks. This memory system is particularly sensitive to age-related memory decline, and older adults often show poorer performance when active maintenance of information is required (Borella et al., 2008; Mammarella et al., 2009). More importantly, studies show that efficient WM functioning supports complex cognitive tasks such as reasoning, language comprehension and learning and ultimately emotion regulation strategies underpinning psychological well-being (Growney & English, 2023).

Accordingly, much research has been done on the effects of WM training used to enhance WM capacity (Jaeggi et al., 2008; Saba & Blanchet, 2021; von Bastian & Oberauer, 2014). Beneficial effects, however, are typically modest and often limited to gains on the trained task (practice effects) and transfer to untrained tasks (near/far transfer) that depend on the same abilities (Jaeggi et al., 2008; Klingberg et al., 2002; see also Morrison & Chein, 2011; Takeuchi et al., 2010). Indeed, many studies have found limited transfer effects after WM training, showing benefits only for tasks that directly resemble the trained task in terms of structure or cognitive demands (e.g., Harrison et al., 2013; Melby-Lervåg & Hulme, 2013; Minear et al., 2016; Soveri et al., 2017). For instance, Minear et al. (2016) examined two forms of working memory training (i.e., spatial n-back and complex verbal span) and found no transfer effects of training on other measures of fluid intelligence and reasoning. These results suggest that WM training may induce changes in the processes and strategies instead of supporting improvements in the general capacity of the working memory system (Melby-Lervåg et al., 2016; Pergher et al., 2020; Sprenger et al., 2013; Redick et al., 2013; von Bastian & Oberauer, 2014). Also, a more recent meta-analysis of working memory training studies adopting a pretest-posttest design found consistent improvements on measures of nearest transfer, such as verbal and visuospatial working memory, but found no substantial evidence of any consistent improvements after working memory training on measures of far transfer cognitive abilities, such as nonverbal and verbal abilities, reading comprehension and arithmetic (Melby-Lervåg et al., 2016).

A possible explanation for these results may be linked to the pervasive presence of WM abilities in everyday cognitive functioning. In fact, WM is used extensively during a wide variety of cognitive tasks, and it may be that the processes involved in WM have been practiced so much that additional practice, or training, yields no effect. Thus, improvements seem to be strongly coupled with the practiced task characteristics. It is possible that during WM training interventions, individuals adopt specific strategies that may be challenging to apply in other circumstances. Hence, transfer effects may occur only if training and transfer task performances engage the same strategies (Gathercole et al., 2019). In contrast, Klingberg et al. (2002) used visuospatial WM tasks such as remembering the position of objects in a 4 × 4 grid or verbal tasks such as remembering phonemes, letters, or digits implemented in a computer program and found improvements in executive functions in children with attentional deficits. Jaeggi et al. (2008) adopted a new and more demanding n-back training paradigm that required participants to carry out two simultaneous tasks. Each task required executive functions to be executed and, since task difficulty was manipulated, this avoided the development of task-specific strategies and/or the employment of automatic processes. Results showed substantial improvements in fluid intelligence in both the control and trained participants.

The extent to which transfer effects are task-specific or process-specific is, however, still a matter of ongoing theoretical and empirical debate. A recent study by Himi et al. (2023) directly investigated the differential effects of content- and operation-specific working memory training interventions, revealing enhanced performance in the trained tasks, but no evidence of a generalization to different tasks adopting the same strategies suggesting that the adoption of specific cognitive strategies alone is unlikely to be responsible for transferring WM training gains across tasks. In addition, some studies have suggested that isolated WM training protocols may improve performance beyond the specific task but only within the confines of the trained cognitive function (Schwaighofer et al., 2015).

Borella et al. (2010) developed a structured and adaptive WM training protocol based on a complex verbal span paradigm—the Categorization Working Memory Span Task (CWMS). This task requires participants to process and store verbal information by categorizing stimuli while simultaneously maintaining target words for subsequent recall, thereby engaging multiple WM mechanisms, including attentional control and updating processes. The authors found robust improvements in the trained task and significant transfer effects to several untrained cognitive domains, including short-term memory, reasoning, inhibitory control, and processing speed. Both the specific gains and the transfer effects were interpreted as consequences of a training procedure that adapted the difficulty of the training task to the participants’ performance while varying the effort of maintenance and processing requirements of the trained task to prevent practice effects. The authors, in line with outcomes from other studies (e.g., Hertzog & Hultsch, 2000; von Bastian & Eschen, 2016), suggested that this training intervention, due to its characteristics, may promote transfer effects by stimulating cognitive flexibility and plasticity, together with interest, since the training task was continuously adapted to create a novel and challenging experience. In addition, the authors suggested that the training schedule, arranged with a fixed interval between sessions, may contribute to the success of the training intervention because it gave participants the time necessary to strengthen the skills acquired and should reduce the risk of losing any beneficial effects of having practiced the task (e.g., Cepeda et al., 2006). Specifically, this training intervention was composed of three 1 h sessions with two-day intervals between sessions. Taken together, this contrasting evidence highlights the need to reconsider the dichotomy between task-specific and process-specific mechanisms and reflect on a new conceptualization of transfer effects that may depend on the complex interplay between task characteristics, cognitive demands, and individual differences.

An interesting meta-analysis by Schwaighofer et al. (2015) systematically examined both near- and far-transfer effects following working memory (WM) training and considered a range of potential moderating variables, such as supervision during training and the duration of training sessions. The authors affirmed that cognitive functions have to be trained for a certain period to generate transfer effects. Interestingly, their results showed that effect sizes due to the length of the training intervention (i.e., total time/number of sessions) were variable, suggesting that the length of training may be a crucial variable for determining near- and far-transfer effects. Notably, these effects appear to be neither consistent nor sufficient to guarantee generalization, particularly in the case of far transfer, which remains inconsistent across studies.

In line with this, Brum et al. (2018) directly investigated the role of training length by examining the effectiveness of the WM training protocol originally developed by Borella et al. (2010) in a Brazilian sample. They specifically manipulated the duration of the intervention by comparing the original version of the protocol to an extended version in which they doubled the number of sessions. Their results indicated that increasing the length of the training did not promote additional benefits on the trained nor near-transfer measures, suggesting that performance improvements in these domains may reach a plateau after a certain level of practice. Nonetheless, the authors argued that extending the training duration may produce some impact on far-transfer measures. Thus, these findings seem to support the idea that training length, in isolation, may not determine far-transfer gains. Rather, its effects should be interpreted in conjunction with the qualitative features of the training protocol, including task complexity, the range of cognitive processes involved, and the outcome measures.

Although training length has been identified as a potentially relevant moderator of transfer gains, with evidence suggesting a positive association consistent with a “longer-is-better” principle (Schwaighofer et al., 2015), the minimal or maximum effective training duration required to produce transfer effects is not yet clear, and further studies are needed to address this issue. In addition, the interpretation of the results is further complicated by the heterogeneity of the WM trainings adopted, which include different task structures and the specific cognitive processes targeted across the various studies. Therefore, it may be difficult to assess whether the observed benefits are attributable to training length per se or to differences in the nature of the interventions used.

Moreover, existing investigations have mainly focused on cognitive outcomes, overlooking wider dimensions of successful functioning that may be critically relevant, especially in aging populations (Cui et al., 2024; Hou et al., 2020). For instance, Brum et al. (2018) did not consider essential aspects related to psychological well-being and subjective health, despite growing evidence that these domains are associated with cognitive functioning. In fact, using a Bayesian network approach, Xu et al. (2025) found a direct link between well-being and working memory function, suggesting that WM may represent a key target for interventions aimed at improving well-being. This gap represents a notable limitation, as it restricts the ecological validity of WM training research and limits our understanding of the extent to which cognitive gains translate into improvements in the quality of life. Together, these considerations highlight two critical gaps in the literature: (1), the need to systematically investigate the optimal duration of WM training required to produce optimal transfer effects; and (2) the importance of adopting a more comprehensive outcome framework that includes indicators of psychological well-being and subjective health.

Thus, we designed a modified version of the Categorization WM Span Task (CWMS; Borella et al., 2010) to directly assess the gains and transfer effects of short and long training interventions. The short intervention included three sessions of training, whereas the long intervention included 18 sessions. Our main aim was to directly investigate how training length may differentially affect transfer effects. We assessed both near- and far-transfer gains by comparing performance on basic cognitive abilities and performance-based tests. In addition, we aimed to expand knowledge by assessing transfer gains on measures considered indices of well-being and subjective health.

## 2. Materials and Methods

### 2.1. Participants

Ninety healthy older adults were recruited from the local community to participate in the study on a voluntary basis. Participants were aged between 59 and 86 years (M = 66.4, SD = 7.5) and reported normal or corrected-to-normal visual and auditory acuity. Exclusion criteria included a history of traumatic brain injury, a diagnosis of neurological or psychiatric disorders, clinically significant symptoms of depression or anxiety, a current diagnosis of substance abuse or dependence, or other known neurological conditions. Older adults were randomized to the study conditions after the baseline assessment. Participants who missed more than 20% of the training sessions, any assessment session, or used only one side of the scale to answer self-report questionnaires were excluded from the analyses. The final sample included eighty-four participants. Demographic and mood information are reported in Table 1. We did not calculate an a priori sample size estimation, but the sample size was comparable to previous studies in the literature (Fu et al., 2020; Borella et al., 2013; Karbach & Verhaeghen, 2014).

The study conformed to the American Psychological Association (APA) ethical standards in the treatment of human research participants and to the provisions of the Declaration of Helsinki principles (World Medical Association, 2013). The study protocol was approved by the Ethics Committee of Psychological Research of the Department of Humanities of the University of Naples Federico II (prot. 16/2025).

### 2.2. Materials

Before and after training, all participants were tested with a battery of tasks designed to assess transfer effects. Transfer refers to the notion that training one cognitive function improves performance on untrained abilities. In line with the Noack et al. (2009), transfer tasks were classified along a continuum from nearest- to far-transfer tasks. Nearest transfer refers to improvements in the trained task or a similar visuospatial task (see Cornoldi & Vecchi, 2003). Near transfer refers to improvements in cognitive abilities or tasks that use cognitive processes similar to those trained. Far transfer occurs when a behavior or strategy learned in one context is successfully applied or leads to improvements in a different context.

The nearest-transfer effect was examined using the criterion task (CWMS) and the Dot Matrix test (see also Miyake et al., 2001), a visuospatial working memory (WM) task that, while differing in material content from the training task, taps the same underlying cognitive construct (i.e., visuo-spatial working memory).

Near transfer effects were assessed using verbal and visuospatial short-term memory measures, including respectively the Forward and Backward Digit Span and the Forward and Backward Corsi Block-Tapping tests (Guariglia, 2007). These tasks are traditionally considered measures of short-term memory, as they differ in processing demands from prototypical WM tasks (Bopp & Verhaeghen, 2005).

Far transfer effects were evaluated through a series of tasks evaluating higher-order cognitive abilities, namely reasoning (Colored Raven’s Progressive Matrices; Raven, 1965), processing speed (Letter Comparison Task and Pattern Comparison task; Salthouse & Babcock, 1991), and inhibitory control (Stroop Color-Word task; Trenerry et al., 1989). These domains were selected based on evidence indicating their association with WM functioning and their role in explaining age-related differences in WM performance when factors such as processing speed and inhibition are considered (Borella et al., 2011).

For the pre- and post-training assessments, parallel versions of each task featuring different stimuli were employed and were counterbalanced across testing interventions to control for practice effects.

In addition, participants completed a set of self-report questionnaires in paper-and-pencil format to assess psychological well-being, including subjective health, emotional and social functioning. Specifically, mood was measured using the Positive and Negative Affect Schedule (PANAS; Watson et al., 1988), subjective feelings of loneliness and social isolation were assessed using the UCLA Loneliness Scale, and perceived health status was evaluated by the 36-Item Short Form Survey (SF-36; Apolone & Mosconi, 1998). From the last questionnaire, we considered only the items of the subscales related to the emotional and social fields and excluded the subscales related to the physical domain.

### 2.3. Procedure

Training sessions in both the short and long interventions were conducted individually and supervised by a trained experimenter. Instructions were identical across training conditions, and no feedback was given during the training sessions.

In the criterion task, participants listened to word lists, each containing five words, with the number of lists per trial varying from three to five. During the processing phase, participants listened to the verbal inputs and were asked to tap their hand on the table whenever they heard an animal noun pronounced by the experimenter. Following the presentation of each trial, during the maintenance phase, participants were instructed to recall the target items from each list in their original serial order. To promote the emergence of generalized transfer effects and limit reliance on task-specific strategies, task demands were systematically manipulated across trials. Maintenance demand was varied by increasing the number of target items (words) to be recalled across the word lists in each trial. To promote the emergence of generalized transfer effects and limit the reliance on task-specific strategies, task demands were systematically manipulated across trials. Specifically, the maintenance demand was adjusted by increasing the number of target items to be recalled across the word lists in each trial. Additionally, task requirements varied across trials, requiring participants to recall either the first or the last target item in each list, or items preceded by an acoustic cue produced by the experimenter. The processing component was also modulated by varying the frequency of animal nouns within the lists, which varied from 0 to 2 in each list, thereby modulating the level of attentional and monitoring demands. The task demand manipulations were identical in the short and long training interventions. The only difference was the number of trials. Specifically, in the short training we created 15 trials: 5 containing 3, 5 containing 4 and 5 containing 5-word lists. In the long training version, we created 90 trials: 30 containing three, 30 containing four and 30 containing five-word lists. The words from the original lists in the short intervention were randomized to create the additional lists in the long version.

## 3. Results

First, to interpret differences between the two groups regarding the training procedure, pretest performance was compared with separate analyses of variance (ANOVAs) conducted for all baseline measures with Group (short vs. long) as the independent variable. Results showed no significant differences between the groups’ baseline performance except for the social role functioning subscale (F_(1,82)_ = 19.038, *p* < .001), and the general health subscale of the SF-36 survey (F_(1,82)_ = 4.3508, *p* = .04), with higher mean scores for the shorter-trained group than the longer-trained group in both cases.

To assess the effects of training, Group (2 levels: short-trained vs. long-trained) × Session (2 levels: pretest and posttest) mixed-design ANOVAs were run for the measures of interest. When significant main effects or interactions were found, Tukey’s correction for multiple comparisons was applied to post hoc comparisons. Table 2 reports the means and standard deviations for all cognitive measures of interest, separately for group and session.

### 3.1. Transfer Effects

#### 3.1.1. Criterion Task: CWMS

Results revealed a significant main effect of session, with posttest performance significantly better than pretest performance for all participants, independent of the length of training (F_(1,82)_ = 85.173, *p* < .001, η^2^p = 0.51).

For the Dot Matrix task, the total number of correctly recalled positions showed a main effect of session, with all participants performing better at posttest than at pretest (F_(1,82)_ = 5.6828, *p* = .02, η^2^p = 0.06). The group × session interaction was also significant (F_(1,82)_ = 10.326, *p* = .002, η^2^p = 0.11). Post hoc comparison showed that performance benefited only after the longer training intervention (*p* < .001).

#### 3.1.2. Near Transfer Effects

We found a significant main effect of session in both the forward (F_(1,82)_ = 12.255, *p* < .001, η^2^p = 0.13) and backward (F_(1,82)_ = 30.7272, *p* < .001, η^2^p = 0.27) Digit Span tests, with better performance at posttest compared to pretest across all participants. The group × session interaction was not significant, suggesting that training length does not affect performance in this task.

We found a main effect of session in both the forward (F_(1,82)_ = 11.052, *p* = .001, η^2^p = 0.12) and backward (F_(1,82)_ = 9.0793, *p* = .003, η^2^p = 0.10) Corsi test showing better performance at posttest compared to pretest in all participants. The group × session interaction was not significant.

#### 3.1.3. Far Transfer Effects

Cognitive tests

Accuracy and mean response times on the Pattern Comparison Task and the Letter Comparison Task, and the interference index for response times in the Stroop Color Task (see Borella et al., 2013), did not reveal any main effects or interactions (all *p* > .05).

On the Colored Raven Matrices Test, the main effect of session was significant, with all participants showing better performance at the posttest compared to the pretest session (F_(1,82)_ = 5.2446, *p* = .02, η^2^p = 0.06). The group × session interaction was not significant.

To assess psychological well-being and subjective health, we carried out Group (2 levels: short-trained vs. long-trained) × Session (2 levels: pretest and posttest) mixed-design ANOVAs for measures of interest. When significant main effects or interactions were found, Tukey’s correction for multiple comparisons was applied to post hoc comparisons. Table 3 reports the means and standard deviations separately for group and session.

UCLA Loneliness Scale scores showed a main effect of session (F_(1,82)_ = 16.223, *p* < .001, η^2^p = 0.17) with all participants showing diminished scores at the posttest compared to the pretest session. The group × session interaction was not significant.

No differences were found in the positive affective score of the PANAS survey, but a main effect of session was found in the negative affective score (F_(1,82)_ = 10.7526, *p* = .002, η^2^p = 0.12), with a significant reduction at the posttest compared with the pretest across all participants independent of the length of the training intervention.

Regarding the selected subscales of the 36-Item Short Form Survey (SF-36; Apolone & Mosconi, 1998) we found a significant group × session interaction in the general health perception subscale (F_(1,82)_ = 5.4361, *p* = .02, η^2^p = 0.06). Post hoc comparisons showed that only the longer-trained group reported higher general health perception at the posttest than at the pretest session (*p* = .05).

## 4. Discussion

An important issue in the cognitive training literature concerns the identification of the optimal training length, that is, the number of sessions and/or the overall duration of the intervention required to maximize training effects. Empirical evidence in this regard is quite divergent. On the one hand, studies have reported that more intensive and prolonged WM training programs seem to be associated with greater benefits in terms of transfer effects (Brum et al., 2018; Schwaighofer et al., 2015). For instance, a meta-analysis by Schwaighofer et al. (2015) showed that the number of sessions and overall training duration are significant moderators of training efficacy, with longer and more intensive interventions generally yielding larger transfer effects. On the other hand, other studies and meta-analyses challenge the robustness of these duration-dependent effects, suggesting that increasing the number of training sessions may not be sufficient to guarantee cognitive improvements (Karbach & Verhaeghen, 2014; Karr et al., 2014). Together, these results underscore the complexity of the duration–response relationship in WM training and suggest that the optimal training outcome may also depend on the interaction of qualitative (e.g., task demands, engagement) and quantitative (e.g., duration, frequency) characteristics. Furthermore, additional and often underexplored dimensions regarding psychological factors such as psychological well-being and subjective health may also be essential for predicting working memory efficiency (Xu et al., 2025).

In our study, we found that all participants generally benefited from the WM training in terms of improved working memory function, independently of the training length and the type of material (verbal or visuospatial). This finding is in line with the meta-analysis by Melby-Lervåg et al. (2016), which also found improvements in verbal and visuospatial working memory nearest transfer measures. Interestingly, training gains seemed to be more pronounced in the long training intervention compared to the short one, suggesting that increasing training time may enhance the degree of improvement in accordance with the longer-the-better principle (Schwaighofer et al., 2015).

Results also suggest that the Borella et al. (2010) WM training intervention, both in its short (three sessions) or long (eighteen sessions) versions, generates near-transfer gains to untrained verbal and visuospatial short-term memory measures, suggesting that both training interventions can stimulate gains in tasks that use cognitive processes similar to those trained. This pattern is contrary to the study by Himi et al. (2023), which found no evidence of transfer between tasks, and this difference may be specifically related to the characteristics of the training protocol adopted. Indeed, the WM training task may stimulate cognitive flexibility and plasticity by systematically varying both processing and maintenance demands, which may subsequently support transfer effects across tasks that use cognitive processes similar to those trained.

In addition, increases in cognitive flexibility and plasticity may also be responsible for the far-transfer gains we found in reasoning ability as measured by the Colored Raven Matrices test. However, considering that we found an improvement in reasoning abilities independent of the training length, an alternative explanation may be related to practice effects. In fact, although we used parallel versions of the task to control for learning effects, we cannot exclude the possibility that task familiarity at posttest may have enhanced performance. Indeed, the idea that WM training may impact other broader cognitive functions, such as fluid intelligence, is quite controversial. While several prior studies have found WM training advantages on measures of fluid intelligence and reasoning abilities (Borella et al., 2010; Jaeggi et al., 2008; Karbach & Kray, 2009), other studies have failed to find these specific improvements (Chein & Morrison, 2010; Dahlin et al., 2008; Harrison et al., 2013). The main explanation across studies for this discrepancy seems to be methodological; in fact, differences in the training protocols are stated as the most probable explanation for these differences. Effectively, both Jaeggi et al. (2008) and Karbach and Kray (2009) employed challenging memory tasks, manipulating demands on goal maintenance or task difficulty, to adapt the task level to each participant’s performance level. Also, Borella et al. (2010, 2013) developed their training program to favor generalized transfer effects by manipulating the maintenance demand of the task, task requirements and the frequency of processing requirements of secondary targets. Such design characteristics are consistent with theoretical accounts that emphasize the importance of variability, adaptivity, and high cognitive load in prompting far-transfer effects in training protocols (Morrison & Chein, 2011; von Bastian & Oberauer, 2014). Moreover, these features may enhance engagement and adherence to the intervention, which have been identified as critical moderators of training efficacy (Jaeggi et al., 2014; Gathercole et al., 2019). From this perspective, the observed far transfer effects may reflect the activation of shared cognitive and neural substrates between working memory and fluid reasoning, supporting the hypothesis of a functional overlap between these constructs (Kane et al., 2005; Engle, 2002). Together, these results extend previous findings by indicating that, although a limited number of sessions may be sufficient to elicit measurable transfer effects, increasing the duration of the intervention appears to amplify at least some of these benefits. This may suggest the existence of a relationship whereby more prolonged engagement in training may facilitate broader generalization processes, especially when transfer involves tasks with partially overlapping but not identical cognitive demands.

More importantly, our findings highlight the potential benefits of WM training on affective functioning and subjective well-being. In general, we found that even a brief intervention consisting of three sessions was associated with a reduction in perceived loneliness and negative affective states. These results are consistent with prior evidence showing that improvements in executive control and working memory capacity can lead to better emotion regulation and reduced vulnerability to negative affect (e.g., Takeuchi et al., 2014).

Furthermore, a noteworthy finding, although debatable since the groups differed in their subjective health mean scores at pretest, with the short-training group showing higher mean scores at pretest than the long-training group, is the improvement we found in general health perception following the long intervention compared to the short one. This is an interesting finding, especially if one considers that we found the opposite pattern at posttest. In other words, even though short-trained participants showed higher scores at pretest, the increment between pre- and post-assessments was greater in the long-trained participants. Furthermore, the mean scores at posttest were quite similar between the two groups, raising the possibility of a regression-to-the-mean effect. This alternative point of view cannot be excluded, and thus, these findings should be interpreted cautiously because we did not perform baseline-adjusted analyses and did not add a control group to the experimental protocol. Nevertheless, such a finding seems to be in accordance with other studies that found that cognitive training interventions may exert wider psychological benefits, including both a reduction in depressive symptoms and improvements in the quality of life (e.g., Lohman et al., 2013; Minihan et al., 2021). Importantly, such outcomes may be particularly relevant in aging populations that often experience concomitant and interconnected declines in cognitive and emotional functioning. From this point of view, working memory training may represent a promising, non-pharmacological and accessible method to promote well-being in aging by simultaneously affecting cognitive efficiency and emotional regulation (Hertzog et al., 2008; Willis et al., 2006). It may therefore be considered a possible target for preventive interventions aimed at improving the overall quality of life in older adults.

Finally, our study is not without limitations. First, we did not include an active control group that would have allowed us to reconfirm the already well-documented gains in the literature. We chose to divide the older adults who participated in the study between training groups in order to include the maximum number of older participants possible, especially since we were specifically interested in examining pre/post training performance following different lengths of intervention. Second, we did not calculate an a priori sample size estimation. However, our sample size is in line with previous studies on cognitive training programs (see Wu et al., 2023 for a systematic review). Finally, we tested participants after three and eighteen sessions of training and found specific gains linked to training length. However, we did not carry out follow-up assessments to determine whether the gains are maintained over time. Also, follow-up assessments would help clarify whether the benefits in psychological well-being were strictly linked to the training protocols or were regression-to-the-mean effects. It would therefore be crucial to examine performance at a three- or six-month follow-up to investigate the duration of the benefits gained from the WM training protocol. Future studies are needed and should address this question.

## Figures and Tables

**Table 1:** Demographic and mood (depression and anxiety) Information for Participants and training length.

Measure	Short Trained		Long Trained		
	Mean	SD	Mean	SD	*p*-Value
Age	66.9	8.3	65.8	6.5	0.35
Education (years)	14.2	3.1	12.6	4.7	0.07
MMSE	28.6	1.7	28.5	1.2	0.71
GDS	7.3	5.3	9.0	6.0	0.19
GAS-12	5.8	3.8	7.6	4.7	0.06

*Note.* MMSE = Mini Mental State Exam (Folstein et al., 1975); GDS = Geriatric Depression Scale (Yesavage et al., 1982); GAS-12 = Geriatric Anxiety Scale-12 (Picconi et al., 2023).

**Table 2:** Means and standard deviations for all cognitive measures, separately for group and training length.

	Short Trained				Long Trained			
Measure	Pretest		Posttest		Pretest		Posttest	
	Mean	SD	Mean	SD	Mean	SD	Mean	SD
CWMS recall	0.6	0.2	0.7	0.2 ***	0.5	0.2	0.7	0.2 ***
Dot Matrix	0.6	0.2	0.6	0.2	0.5	0.2	0.7	0.2 ***
Forward Digit	6.8	1.9	7.2	2.3 ***	6.1	2.1	6.8	2.1 ***
Backward Digit	6.1	2.3	7.2	2.5 **	5.6	2.1	6.4	2.4 ***
Forward Corsi	5.7	1.3	6.1	1.4 **	5.5	1.7	6.3	2.0 ***
Backward Corsi	5.9	1.7	6.2	2.1 **	5.7	1.8	6.6	2.2 **
Pattern Comp.	98.4	28.3	92.7	27.4	93.9	35.7	101.2	41.6
Letter Comp.	96.0	34.4	101.4	27.9	110.9	54.6	119.4	65.9
Stroop Index	0.7	0.3	0.6	0.4	0.5	0.3	0.5	0.4
Raven Matrices	0.7	0.2	0.8	0.1 *	0.7	0.2	0.8	0.2 *

Note: * *p* < .05, ** *p* < .01, *** *p* < .001. CWMS = Categorization Working Memory Span Task; Dot Matrix (Miyake et al., 2001); Forward and Backward Digit Span (Mondini et al., 2011); Forward and Backward Corsi Test = Corsi Block-Tapping Task (Corsi, 1972); Pattern comp. = Pattern Comparison Task (Salthouse & Babcock, 1991); Letter comp. = Letter Comparison Task (Salthouse & Babcock, 1991); Stroop Index = Stroop Interference Index (Stroop, 1935); Raven Matrices = Colored Raven Matrices Test (Raven, 1965).

**Table 3:** Means and standard deviations for all measures of psychological well-being, separately for group and training length.

	Short Trained				Long Trained			
Measure	Pretest		Posttest		Pretest		Posttest	
	Mean	SD	Mean	SD	Mean	SD	Mean	SD
UCLA	18.8	3.7	17.2	3.7 ***	19.5	5.8	18.1	4.8 ***
PANAS Positive	31.9	6.5	32.1	5.8	29.0	7.5	28.5	7.4
PANAS Negative	17.8	4.3	16.8	4.9 **	19.0	6.4	17.3	6.1 **
SF-36—Vitality	61.4	17.3	62.9	16.7	56.5	16.9	57.1	16.7
SF-36—Emotional well-being	69.7	14.8	72.2	14.9	64.1	17.7	64.1	19.0
SF-36—Social functioning	86.3	14.0	87.5	17.5	71.4	17.1	72.9	25.6
SF-36—General Health	65.0	15.8	62.6	15.3	57.6	16.6	62.1	15.9 *
SF-36—Role Emotional limits	23.8	33.2	28.8	29.7	16.7	26.8	17.5	21.1

Note: * *p* < .05, ** *p* < .01, *** *p* < .001. UCLA = University of California, Los Angeles Loneliness Scale (Russell et al., 1978); PANAS = Positive and Negative Affect Schedule (Watson et al., 1988); SF-36 = Short Form Health Survey 36 (Ware & Sherbourne, 1992).

## Data Availability

The data presented in this study are available on request from the corresponding author according to privacy standards.
